# 

**Published:** 1818-04

**Authors:** 


### CORRESPONDENCE.

Mr. Cunningham's Observations on the Administration of Calomel in Scruple Doses, with Cases, will appear in our next Number.

A Review of Dr. Marcet's Essay on the Chemical History and Medical Treatment of Calculous Disorders will also appear in our ensuing publication.

Mr. Home Blackadder's Observations on Phagedæna Gangrænosa; Dr. Veitch on the non-contagious Nature of the Yellow Fever, &c.; and Dr. J. Walker's Reply to Mr. Moore, on Vaccination, have been received, and will be early attended to.

---

☞ Authors and Publishers are requested to send Copies of new Works for Review as early as possible after their publication. They will be reviewed early in proportion.

